# Genome-Wide Association Studies Provide Insights Into the Genetic Architecture of Seed Germination Traits in Maize

**DOI:** 10.3389/fpls.2022.930438

**Published:** 2022-06-10

**Authors:** Yuntong Li, Yameng Liang, Meiling Liu, Qiyuan Zhang, Ziwei Wang, Jinjuan Fan, Yanye Ruan, Ao Zhang, Xiaomei Dong, Jing Yue, Cong Li

**Affiliations:** ^1^College of Bioscience and Biotechnology, Shenyang Agricultural University, Shenyang, China; ^2^State Key Laboratory of Plant Physiology and Biochemistry, National Maize Improvement Center, Key Laboratory of Biology and Genetic Improvement of Maize (MOA), Beijing Key Laboratory of Crop Genetic Improvement, China Agricultural University, Beijing, China; ^3^College of Pharmaceutical and Biological Engineering, Shenyang University of Chemical Technology, Shenyang, China

**Keywords:** genome-wide association study, germination speed, germination consistency, molecular breeding, maize

## Abstract

Seed germination is an important agronomic trait that affects crop yield and quality. Rapid and uniform seed germination traits are required in agricultural production. Although several genes are involved in seed germination and have been identified in Arabidopsis and rice, the genetic basis governing seed germination in maize remains unknown. Herein, we conducted a genome-wide association study to determine the genetic architecture of two germination traits, germination speed, and consistency, in a diverse panel. We genotyped 321 maize inbred populations with tropical, subtropical, or temperate origins using 1219401 single-nucleotide polymorphism markers. We identified 58 variants that were associated with the two traits, and 12 of these were shared between the two traits, indicating partial genetic similarity. Moreover, 36 candidate genes were involved in seed germination with functions including energy metabolism, signal transduction, and transcriptional regulation. We found that favorable variants had a greater effect on the tropical subpopulation than on the temperate. Accumulation of favorable variants shortened germination time and improved uniformity in maize inbred lines. These findings contribute significantly to understanding the genetic basis of maize seed germination and will contribute to the molecular breeding of maize seed germination.

## Introduction

As the primary food source, crops have supported human development and progress over several 1,000 years. The life cycle of the majority of crops begins with seed germination and continues to juvenile and adult vegetative stages, reproductive stages, and seed production. Seed vigor is an important agricultural trait that affects crop yield and quality directly and indirectly (Tekrony and Egli, 1991; Ellis, 1992; Larsen et al., 1998). Seed germination is an important component of seed vigor, which is defined as the potential activity of the seeds (Perry, 1978; Association of Official Seed Analysis, 1983).

The process of seed germination can be categorized into three physiological phases from the mature dry seed to the protrusion of the radicle. During the initial phase (Phase I), dry seeds rapidly absorb water through physical absorption until the seed’s cell is hydrated. When the water content of the seed is maintained at a constant level, physical imbibition is complete (Phase II, plateau phase). The protrusions of the radicle and coleoptile then break through the testa, and the growth becomes visible (Phase III; Bewley, 1997; Nambara et al., 2010; Nonogaki et al., 2010). Seed germination occurs strictly in Phases I and II, during which the seed undergoes complex physiological and biochemical changes (Rajjou et al., 2012). According to transcriptome data, dry seeds of Arabidopsis and barley contain over 12,000 transcripts, whereas those of rice contain over 17,000 transcripts (Nakabayashi et al., 2005; Howell et al., 2008; Sreenivasulu et al., 2008). Extant mRNAs stored in seeds are directly used to guide *de novo* protein synthesis during the early germination stage *via* seed imbibition and cell rehydration (Kimura and Nambara, 2010). Moreover, during germination, the damage to DNA and proteins caused by oxidative stress during the seed dehydration, storage, and rehydration stages are repaired (Cheah and Osborne, 1978; Dandoy et al., 1987; Oge et al., 2008). Subsequently, new genes are transcribed and they continue to participate in the regulation of germination (Howell et al., 2008). Glycolysis and tricarboxylic acid (TCA) cycle are then activated to provide energy for seed germination, and normal metabolism is gradually restored following imbibition (Hourmant and Pradet, 1981; Howell et al., 2008).

Plant hormones, particularly abscisic acid (ABA) and gibberellin (GA), are critical for the regulation of seed germination. A high ABA concentration promotes dormancy and inhibits seed germination, whereas high GA levels promote seed germination by reversing dormancy. The ratio of ABA to GA has a significant effect on the metabolic transition for seed germination (Yamaguchi, 2008; Nambara et al., 2010). Several genes regulate seed germination by regulating ABA biosynthesis or signal transduction. The enzyme 9-cis-epoxycarotenoid dioxygenase (NCED) catalyzes 9-cis neoxanthin to xanthoxin and is involved in the biosynthesis of ABA, which regulates seed germination (Bassel et al., 2008). ABA INSENSITIVE 3 (*ABI3*) and *ABI5*, which act downstream of *ABI3*, have a significant effect on seed germination in ABA signaling (Lopez-Molina et al., 2001). Additionally, the level of GA is regulated by the GA biosynthesis genes, such as *GA20ox3*, *GA3ox1*, and *GA2ox5*, which are required for seed germination (Yamauchi et al., 2004; Iglesias-Fernandez and Matilla, 2009).

Posttranslational protein modifications (PTMs) are critical for seed germination control as they can affect the activity and stability of proteins (Rajjou et al., 2012). Phosphorylation and dephosphorylation of proteins are critical regulatory mechanisms. Many phosphatases and kinases are involved in seed germination processes, including energy metabolism, DNA repair, and phytohormone signaling pathway (Hourmant and Pradet, 1981; Brock et al., 2010; Hubbard et al., 2010). Three members of the sucrose non-fermenting 1-related protein kinase 2 (SnRK2) in Arabidopsis, SnRK2.2, SnRK2.6, and SnRK2.3, play important roles in the phosphorylation of ABI5 to influence seed development and germination (Nakashima et al., 2009). Mitogen-activated protein kinases (MAPKs) also play a role in regulating seed germination. MAPK11 interacts with phosphorylate SnRK1, whereas protein phosphatase 2C (PP2C) promotes seed germination in tomatoes by inhibiting SnRK2.2, thus activating ABI5 and inhibiting germination (Song et al., 2021). Similarly, Arabidopsis PP2C5 promotes seed germination through direct interaction with MAPK3, MAPK4, and MAPK6, all of which are involved in ABA signaling (Brock et al., 2010). Other PTMs, such as glycosylation, ubiquitination, and acetylation, play a role in seed germination (Arc et al., 2011). Additionally, certain cell wall remodeling enzymes, such as xyloglucan endotransglycosylase/hydrolase and cellulose, may play a significant role in the germination process as water uptake results in increased cellular volume and turgor pressure in dry seeds (Nonogaki et al., 2010).

Germination is a complex quantitative trait that is regulated by a large number of genetic loci and is influenced by environmental factors. Quantitative trait locus mapping and genome-wide association study (GWAS) are two critical methods in quantitative genetics that are widely used to dissect the genetic architecture of seed germination in plants (Schaar et al., 1997; Anzala et al., 2006; Ikeda et al., 2009; Landjeva et al., 2010; Jiang et al., 2011; Xie et al., 2014). The Delay of Germination 1 (*DOG1*) gene is a critical regulator of seed dormancy and germination, and it was positionally cloned from Arabidopsis (Bentsink et al., 2006). *DOG1* regulates seed dormancy and inhibits germination by interacting with and inhibiting the activity of two PP2Cs, AHG1 and AHG3 (Née et al., 2017). In rice, seed dormancy 4 (*Sdr4*) was cloned using positional cloning and acted downstream of *OsVP1* to inhibit seed germination (Sugimoto et al., 2010). GWAS has also been used to dissect the genetic basis of rice (Shi et al., 2017), sorghum (Upadhyaya et al., 2016), and rape seed (Hatzig et al., 2015) germination traits. However, there are only a few studies on the genetic basis of maize seed germination (ref), and studies on maize seed germination are even more scarce.

In this study, we used 1,219,401 single-nucleotide polymorphisms (SNPs) and a mixed linear model (MLM) to conduct a GWAS on two seed germination traits, germination speed (GS), and germination consistency (GC), in a maize association panel comprising 321 diverse inbreds. We identified a series of significant SNPs and 36 candidate genes associated with GS and GC. These two traits partially share a genetic basis. Proper accumulation of favorable alleles may result in a reduction in germination time or an increase in germination uniformity. These results provide available resources that could be used to enhance maize seed germination.

## Materials and Methods

### Plant Materials

An association panel comprising 508 diverse maize inbred lines (Liu et al., 2017) was grown in Tieling City, Liaoning Province, China (42.5°N, 124.2°E) in May 2016. To minimize the environmental influence, seeds from 321 maize inbreds that were simultaneously pollinated were selected to evaluate seed germination phenotypes. Based on the population structure (membership probabilities ≥0.60), the 321 maize accessions were categorized into 72 tropical lines, 140 temperate lines (NSS and SS), and 109 mixed lines (Yang et al., 2011; Li et al., 2013). All seeds were dried and stored for at least 6 months to break dormancy.

### Seed Germination Test and Phenotype Assessment

We used 10-cm square plastic petri plates filled with vermiculite for the germination test. Each petri plate with vermiculite was filled with 80 ml of distilled water. The seeds of each accession were sterilized for 10 min with a 1% sodium hypochlorite solution and then rinsed four times with distilled water. Subsequently, 20 seeds from each accession were placed in a petri plate filled with moist vermiculite and the embryo was kept upright to observe germination. All petri plates were placed in a phytotron set to 28°C for dark conditions with a relative humidity of 100%. When the radicles and coleoptiles reached a visible length of ≥2 mm, the seeds were considered to have germinated. Every 4 h, the number of germinated seeds was counted. The time required for 50% of seeds to germinate represents the GS. The first germination of a seed is denoted by the term first germination (FG). The formula used to calculate GC is as follows: GC = GS − FG. Using MLM, we calculated the best linear unbiased prediction (BLUP) values for four independent germination experiments. For each trait, the based broad-sense heritability was calculated as $h^{2}=\sigma_{g}^{2}/\left(\sigma_{g}^{2}+\sigma_{e}^{2}/n\right)$, where $\sigma_{g}^{2}$ is the genetic variance, $\sigma_{e}^{2}$ is the residual error variance, and n represents the number of replications (Coles et al., 2010).

### Genome-Wide Association Analysis

The BLUP values and 1,219,401 SNPs with a minor allele frequency of ≥0.05 in 321 lines (Liu et al., 2017) were used to conduct GWAS, which was implemented using an MLM in TASSEL V5.0 (Bradbury et al., 2007), where the population structure matrix (*Q*) and the relative kinship matrix (*K*) matrices were fitted to control spurious associations. To avoid false negatives, we used a threshold of *p* ≤ 1.82 × 10^−5^ (1/54926) for determining significant SNPs. A 54,926 is the number of independently occurring SNPs (*r*^2^ < 0.1) as determined by Plink v1.07. The R function lm () was used to determine the explained phenotypic variance of mutual SNPs in GS and GC.

### Candidate Genes Annotation

The most significant SNP within an LD block comprising adjacent significant SNPs with *r*^2^ > 0.1 of each other was chosen to represent the locus associated with GC or GS. The physical locations of significant SNPs were determined using the B73 RefGen v2 database. Candidate genes were annotated using data from the maize GDB website. The Maize GDB Gene Function and Expression database were used to download raw RNA-seq and proteomics data (https://download.maizegdb.org/ GeneFunction and Expression).

### RNA Extraction and RT-qPCR

The seeds of B73 were sterilized with 1% sodium hypochlorite solution for 10 min, rinsed four times with distilled water, and then transferred to 9-cm plastic petri plates lined with wet filter paper in a growth chamber set to 28°C for imbibition. Embryo and endosperm were isolated from dry seeds or seeds at various stages of imbibition and stored at −80°C. The RNeasy Plant Mini kit was used to extract the RNA (Qiagen). cDNA was synthesized from 2 μg of total RNA using the M-MLV reverse transcriptase (Promega). RT-qPCR reactions were performed using an ABI7500 real-time PCR system using the SYBR Premix Ex Taq II kit (Takara). Transcript levels were analyzed using the comparative CT (2^−△CT^) method (Schmittgen and Livak, 2008). *ZmTubulin1* (GRMZM2G152466) was used as an internal control for data normalization. All data were measured in three independent biological replicates. The primers are listed in Supplementary Table S1.

### Allele Pyramiding

Plink v1.07 was used to determine the independent significant SNPs with parameter --no-parents --no-sex --no-fid --*r*^2^ --ld-window 1 --ld-snp-list --ld-window-*r*^2^ 0. If *r*^2^ between two SNPs is less than 0.1, the two SNPs are considered as independent. To avoid the effect of missing values, independent significant SNPs were further filtered with missing rate less than 10% in each maize subgroup (temperate maize or tropical maize), and the resulting SNPs were used for allele pyramiding.

## Results

### Characterization of Maize Seed Germination Traits

Environmental factors that occur during seed maturation can affect seed germination. To mitigate the effects of environmental heterogeneity during maturation, 321 diverse maize inbred lines harvested at similar times were used to estimate germination traits. These lines were selected from an association panel of 508 accessions that have been widely used to dissect the genetic architecture of complex traits such as oil content, drought tolerance, and flowering time (Li et al., 2013; Liu et al., 2013; Guo et al., 2018a).

**Figure 1:**
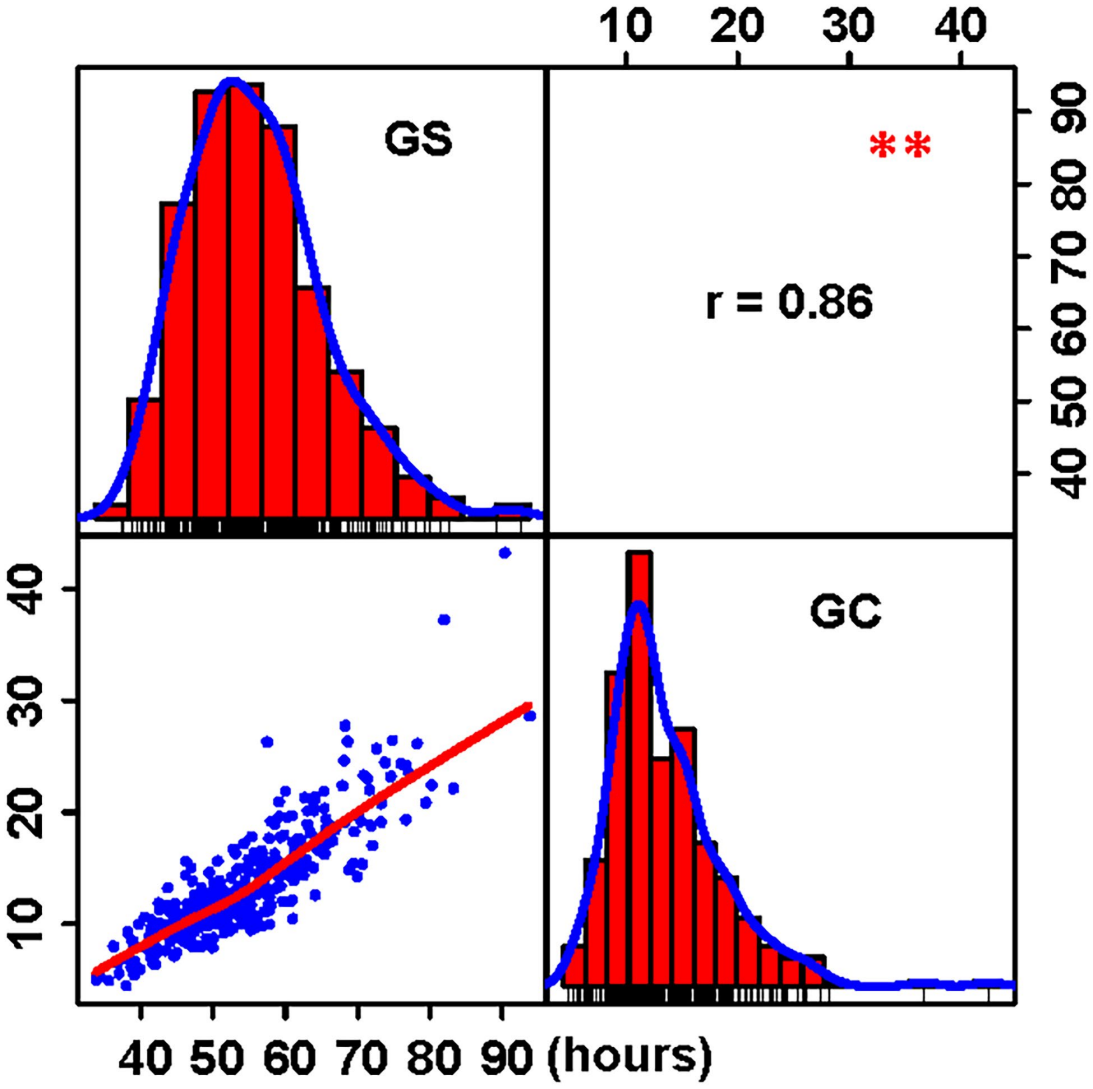
Frequency distributions and correlations of two germination traits. The plots on the diagonal show the phenotypic distribution of two traits. The plots below the diagonal line are the scatter plots compared with two traits, and the values above the diagonal line are Pearson correlation coefficient between traits. ^**^p ≤ 0.01; GS represents the germination speed; and GC represents the germination consistence.

The time taken for 50% of the seeds to germinate represents seed GS, and the time interval between the first and 50% seed germination represents GC. Four independent germination experiments were conducted to determine the effect of the environment on germination. Following that, the phenotypic BLUP data from four germination tests were calculated and analyzed. Both GS and GC in our panel had a near-normal distribution (Figure 1) and a wide range of values, ranging from 33.78 to 93.67 h (mean 53.99 ± 10.19 h) for GS and 4.42 to 43.21 h (mean 13.37 ± 5.12 h) for GC (Table 1). Additionally, GS and GC had a strong positive correlation (*r* = 0.86; Figure 1). The broad-sense heritability (*h^2^*) was then calculated using the method of Coles et al., (2010). GS had a higher heritability (*h^2^* = 0.75), whereas GC had a moderate heritability (*h^2^* = 0.58; Table 1). These findings indicate that the population’s broad phenotypic variations in GS and GC are largely a result of genetic factors and thus are suitable for further association mapping.

**Table 1:** Phenotypic performance, variance component, and broad-sense heritability of germination speed (GS) and germination consistency (GC).

Trait	Range (h)	M ± SD (h)	*h*^2^^a^
GS	33.78–93.67	53.99 ± 10.19	0.75
GC	4.42–43.21	13.37 ± 5.12	0.58

a Family mean-based broad-sense heritability.

### Genome-Wide Association Analysis of GS and GC in Maize

**Figure 2:**
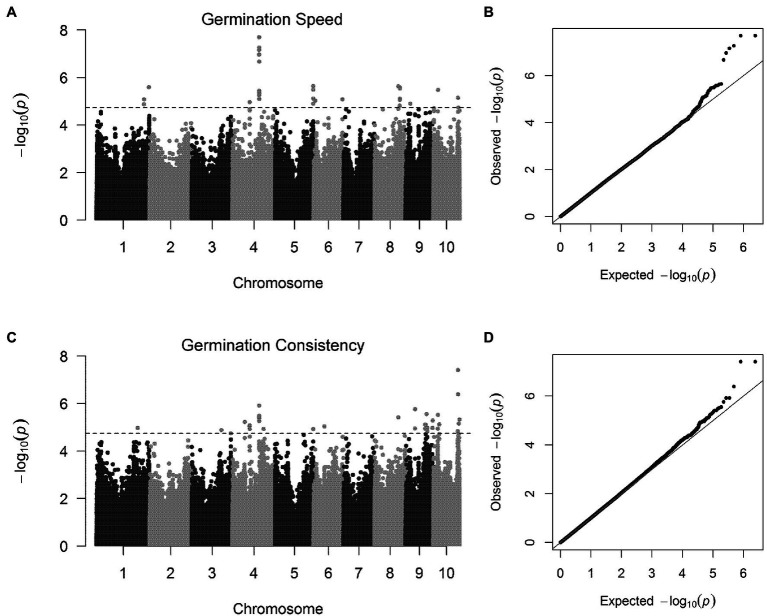
Genome-wide association mapping of GS and GC using MLM method. The Manhattan plots show significant *p*-values associated with GS and GC from a genome-wide scans plotted against the position on each of the 10 chromosomes. Genomic locations and allele effects of significant SNPs located representative genes for GS and GC. The dashed line indicates the genome-wide significance thresholds of 1.82 × 10^−5^. The vertical axis in quantile–quantile plots represents observed *p*-values and the horizontal axis indicates expected values. **(A,B)** are the Manhattan plot and quantile–quantile plot for GS, respectively. **(C,D)** are the Manhattan plot and quantile–quantile plot for GC, respectively.

To ascertain the genetic basis of GS and GC, we conducted a GWAS on the two traits in a panel of 321 inbreds using BLUP data as the phenotype and a MLM as the genotype. A 33 significant SNPs for GS were identified on chromosomes 1, 4, 5, 6, 8, 9, and 10 (Figures 2A,B; Table 2). A 37 significant SNPs in nearly 10 chromosomes, except for chromosomes 2 and 7, were identified for GC (Figures 2C,D; Table 2).

**Table 2:** SNP chromosomal positions and candidate genes are significantly associated with germination traits identified by GWAS.

Trait	SNP	Chr	Position (bp)	R^2^ (%)^a^	*p*-value	Gene	Annotation
GS	chr1.S_268251922	1	268251922		1.34E-05	GRMZM5G881950	Unknown
GS	chr1.S_268736499	1	268736499	6.1	8.34E-06	GRMZM2G137788	Receptor protein kinase TMK1 precursor
GS	chr1.S_296259764	1	296259764	7.4	2.59E-06	GRMZM2G113137	Cellulose synthase
GS	chr4.S_96832829	4	96832829	6.3	1.08E-05	GRMZM5G835747	Unknown
GS	chr4.S_150576794	4	150576794	9.8	2.04E-08	GRMZM2G163193	FTSH protease
GS	chr5.S_215481320	5	215481320	6.7	3.26E-06	GRMZM5G841101	RNA-binding (RRM/RBD/RNP motifs) family protein
GS	chr5.S_21550332		215505332		1.27E-05	GRMZM2G379002	Unknown
GS	chr5.S_216179154		216179154		2.30E-06	GRMZM2G155699	AT-hook motif nuclear-localized protein
GS	chr6.S_9491447	6	9491447	7.6	9.59E-06	GRMZM2G301884	P-loop containing nucleoside triphosphate hydrolases
GS	chr6.S_164142777	6	164142777	6.2	8.39E-06	GRMZM2G377615	RHO guanyl-nucleotide exchange factor 12
GS	chr8.S_133947794	8	133947794	12.6	2.39E-06	GRMZM2G141859	Protein phosphatase 2C
GS	chr8.S_141651532	8	141651532		1.55E-05	GRMZM2G351990	Unknown
GS	chr8.S_143468126	8	143468126	7.5	2.65E-06	GRMZM2G059893	ARM repeat domain protein kinase
GS	chr9.S_26606351	9	26606351	5.8	1.28E-05	GRMZM5G870176	Cellulose synthase-like D3
GS	chr10.S_26483340	10	26483340	7.0	3.33E-06	GRMZM2G162972	Unknown
GS	chr10.S_140984524	10	140984524	6.1	7.22E-06	GRMZM2G129133	Calcium-binding EF hand family protein
Total^b^							
GC	chr1.S_232744843	1	232744843	11.822	1.08E-05	GRMZM2G167438	3-ketoacyl-CoA synthase
GC	chr3.S_168961869	3	168961869	6.982	1.33E-05	GRMZM5G882078	Serine/threonine protein kinase
GC	chr4.S_69283020	4	69283020	10.93	6.01E-06	GRMZM2G133941	Unknown
GC	chr4.S_96832829	4	96832829	6.302	8.56E-06	GRMZM5G835747	Unknown
GC	chr4.S_150576794	4	150576794	9.83	1.22E-06	GRMZM2G163193	FTSH protease
GC	chr4.S_176501405	4	176501405	6.763	1.19E-05	GRMZM2G091189	Chaperonin
GC	chr5.S_216179154	5	216179154	7.258	1.20E-05	GRMZM2G155699	DNA binding protein
GC	chr6.S_62450201	6	62450201	8.075	9.37E-06	GRMZM2G444845	BAG domain containing protein
GC	chr8.S_96,269452	8	96269452	7.48	2.14E-06	GRMZM5G821139	Unknown
GC	chr8.S_133947794	8	133947794	12.618	3.90E-06	GRMZM2G141859	Protein phosphatase 2C
GC	chr9.S_53552857	9	53552857	12.633	1.75E-06	GRMZM5G802679	Unknown
GC	chr9.S_111117600	9	111117600		7.83E-06	GRMZM2G465383	Unknown
GC	chr9.S_114102553	9	114102553		7.83E-06	GRMZM2G095219	Unknown
GC	chr9.S_119304980	9	119304980	15.133	2.82E-06	GRMZM2G098079	Transferases transferring acyl groups
GC	chr9.S_123213461	9	123213461	6.464	1.08E-05	GRMZM2G109286	Phospholipase C
GC	chr9.S_124319815	9	124319815	6.386	1.50E-05	GRMZM2G163774	Cytochrome P450
GC	chr9.S_152678737	9	152678737	5.957	1.08E-05	GRMZM2G063387	Transcription factor DP
GC	chr9.S_154644180	9	154644180	10.004	5.29E-06	GRMZM2G109987	Leucine zipper family protein
GC	chr9.S_26358058	10	26358058		1.15E-05	GRMZM2G130724	Unknown
GC	chr10.S_26483340	10	26483340	6.955	3.07E-06	GRMZM2G162972	Unknown
GC	chr10.S_34368108	10	34368108	8.286	7.71E-06	GRMZM2G119465	Phosphatidylinositol transfer family protein
GC	chr10.S_138881361	10	138881361	6.545	1.48E-05	GRMZM2G091579	Unknown
GC	chr10.S_140984,524	10	140984524	6.145	3.96E-08	GRMZM2G129133	Calcium-binding EF hand family protein
GC	chr10.S_145197,460	10	145197460	7.894	7.08E-06	GRMZM2G086030	Tetratricopeptide repeat (TPR)-like superfamily protein
GC	chr10.S_149450922	10	149450922	10.331	4.73E-06	GRMZM5G869572	Transportin
Total^b^							

a Percentage of phenotypic variation explained by the additive effect of the single significant SNP.

b Total percentage of phenotypic variation explained by all the significant SNPs.

**Figure 3:**
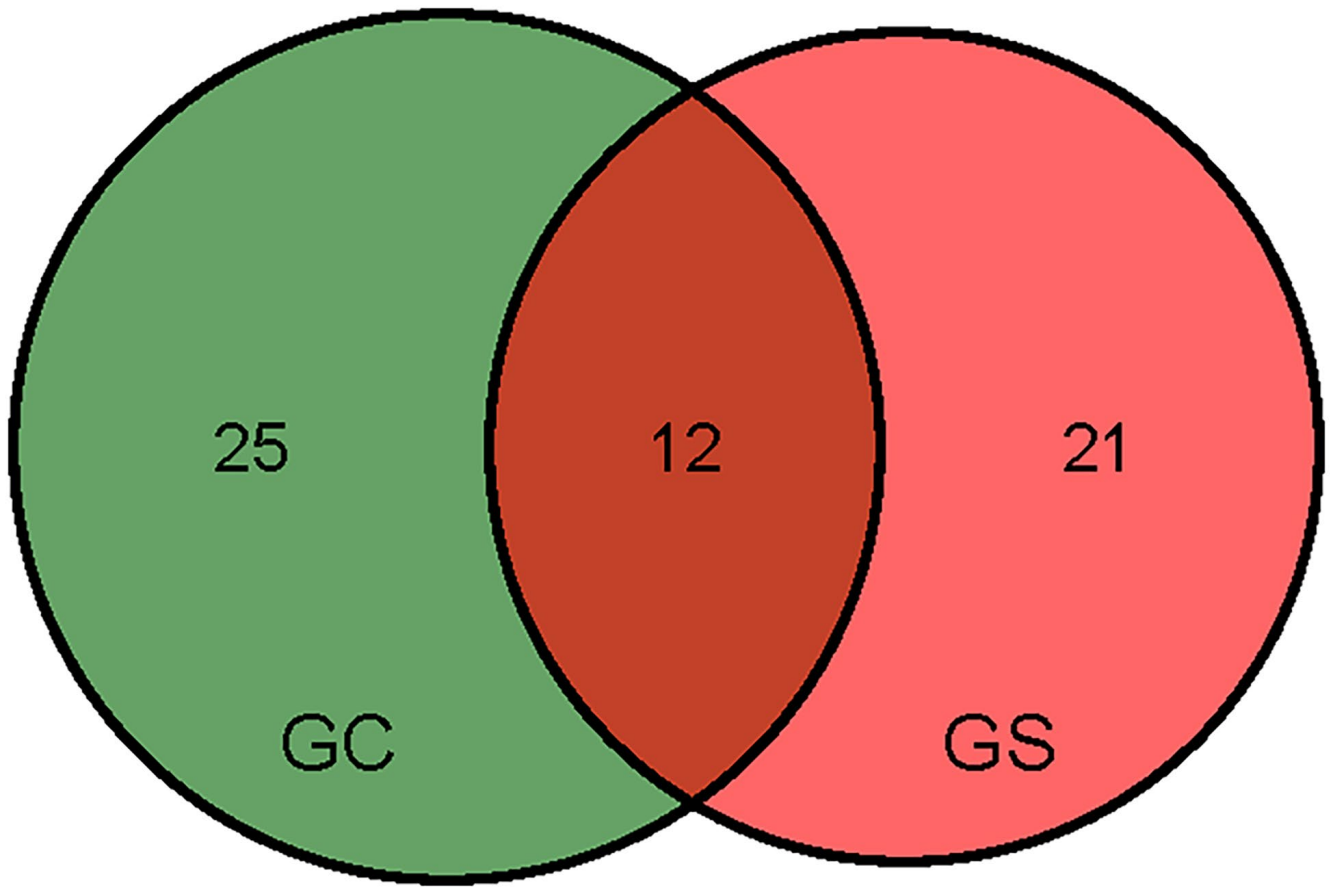
Venn diagram showing the SNP overlap between GC and GS.

To better understand the genetic basis of GS and GC, we analyzed the significant SNPs for GS and GC. Twelve SNPs were detected in both GS and GC (Figure 3). The common SNPs account for 27.8% of GS phenotypic variation and 23.8% of GC phenotypic variation, and both of the most significant SNPs for GS (chr4.S_150576794) and GC (chr10.S_140984524) are included in the common SNPs (Table 2). These findings indicate that GS and GC in maize are controlled by multiple genetic loci and that the two germination traits have a genetic basis that is partially overlapped, which is consistent with the high correlation between GS and GC.

### Candidate Genes Identification

A total of 58 SNPs were identified that were significantly associated with two germination traits, 46 of which are located within the gene body regions, 1 of which is located 2 kb upstream of the coding region, and the remaining 11 of which are located 300 bp–5 kb downstream of the coding genes. A total of 36 candidate genes were identified that co-localized significantly with SNPs, including 16 genes for GS, 25 genes for GC, and 6 genes for both traits. The molecular functions of the candidate genes were found to be involved in a variety of categories, including energy metabolism, signal transduction, and transcriptional regulation (Table 2).

**Figure 4:**
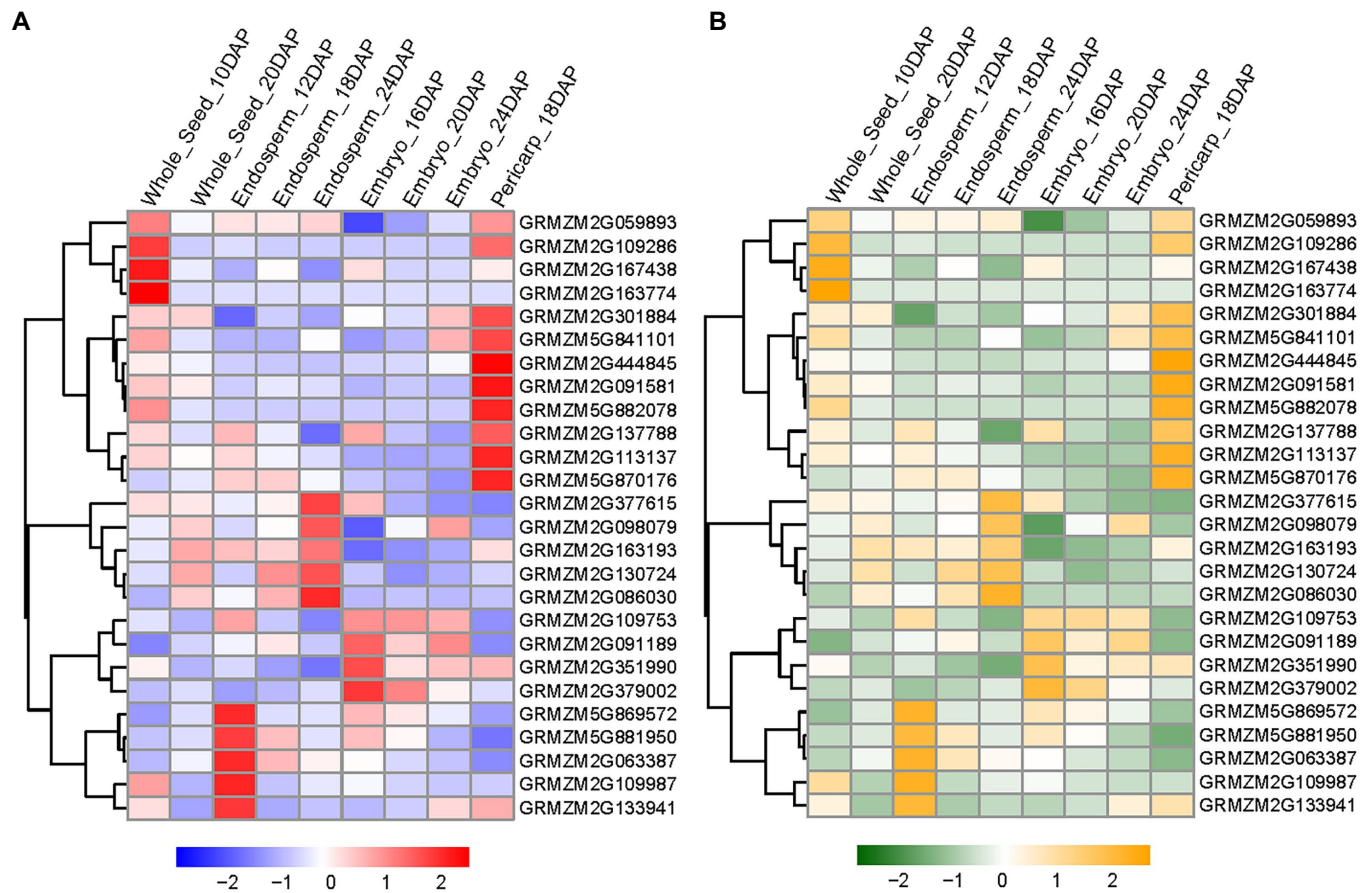
Expression and protein accumulation profiles of candidate genes identified by GWAS. **(A)** The heat map exhibits expression patterns of candidate genes during seed development. Log2-based fold change is used to create the heat map and fold changes are shown in color. **(B)** The heat map exhibits protein accumulation of candidate genes during seed development. Log2-based fold change is used to create the heat map and fold changes are shown in color.

To ascertain which candidate is more likely to be responsible for the significant SNP, we performed additional analysis on their expression and protein pattern using previously published RNA-seq and proteomics data from different seed development stages (Sekhon et al., 2011; Stelpflug et al., 2016). We noted that 26 of the 36 genes are expressed during seed development (Figure 4A). Furthermore, proteins encoded by these 26 genes were accumulated during the seed development and germination stage (Figure 4B).

**Figure 5:**
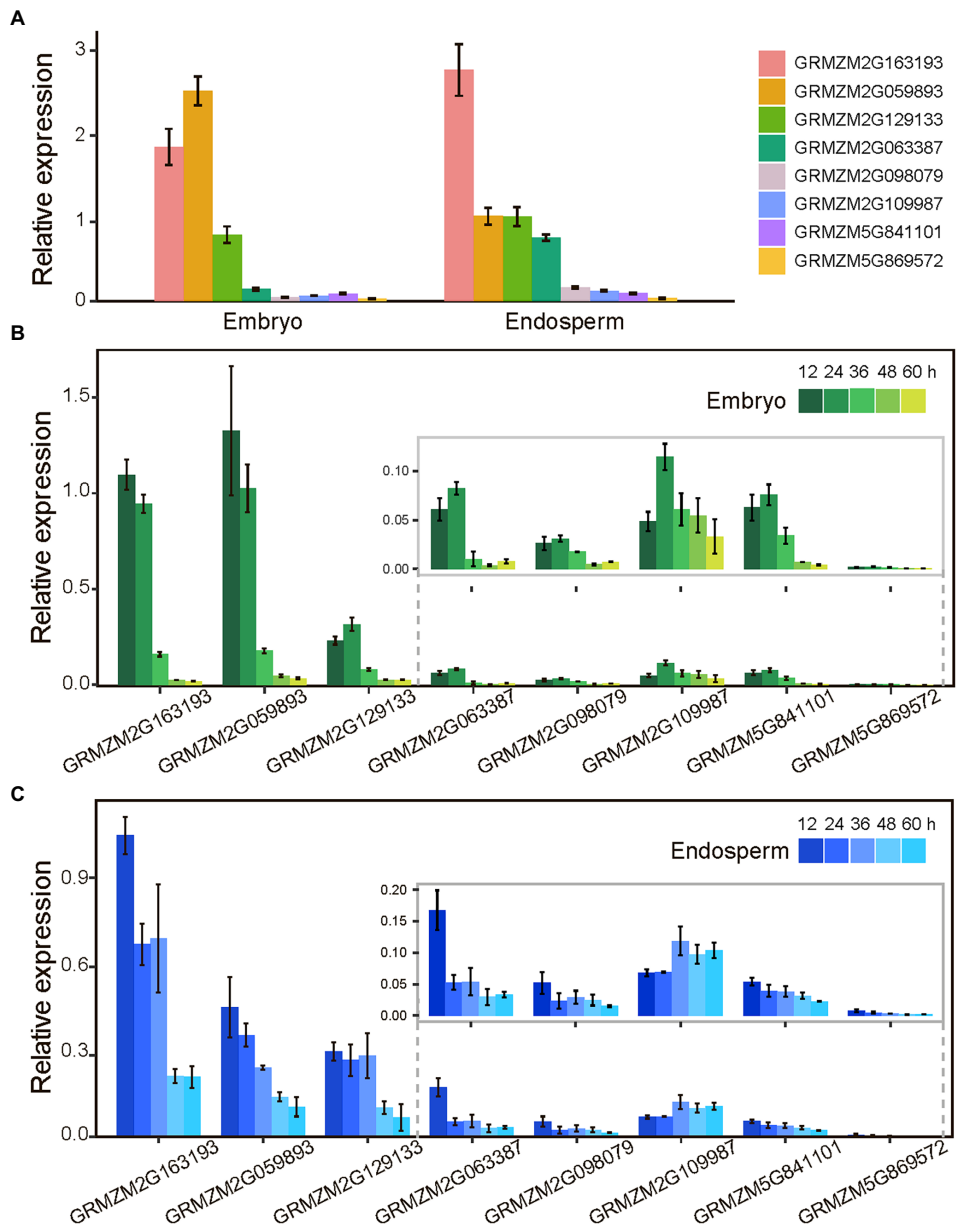
Expression patterns of candidate genes in dry and germination seed by qRT-PCR. **(A)** The expression levels of candidate genes in dry embryo and endosperm. **(B)** Expression levels of candidate genes in the embryo of imbibition seeds. **(C)** Expression levels of candidate genes in the endosperm of imbibition seeds. Vertical bars indicate standard deviation.

According to the functions and expression patterns of genes and the significance of SNPs, eight genes were selected for further expression pattern analysis in dry and imbibing seeds using qRT-PCR, including two candidate genes for GS (GRMZM5G841101 and GRMZM2G059893), four candidate genes for GC (GRMZM2G098079, GRMZM2G063387, GRMZM2G109987, and GRMZM5G869572), and two candidate genes for both traits (GRMZM2G163193 and GRMZM2G129133). In dry endosperm, four genes (GRMZM2G163193, GRMZM2G059893, GRMZM2G129133, and GRMZM2G063387) had significantly higher transcriptional levels than others, and three were also highly expressed in dry embryo (GRMZM2G163193, GRMZM2G059893, and GRMZM2G129133; Figure 5A). The presence of transcripts from eight genes in dry seeds indicated that they had been stored and prepared for seed germination under optimal conditions. During the seed imbibition stage, the expression patterns of eight genes were also detected. Within 24 h of imbibition, the transcriptional levels of six genes increased in the embryo and then decreased. During the imbibition stage, the expression levels of the other two genes (GRMZM2G163193 and GRMZM2G059893) continued to decline (Figure 5B). Except for GRMZM2G109987, the transcriptional levels of all genes decreased during seed imbibition (Figure 5C). The fact that the transcriptional levels of eight genes varied in response to seed imbibition time suggest that these candidate genes may be involved in seed germination processes.

**Figure 6:**
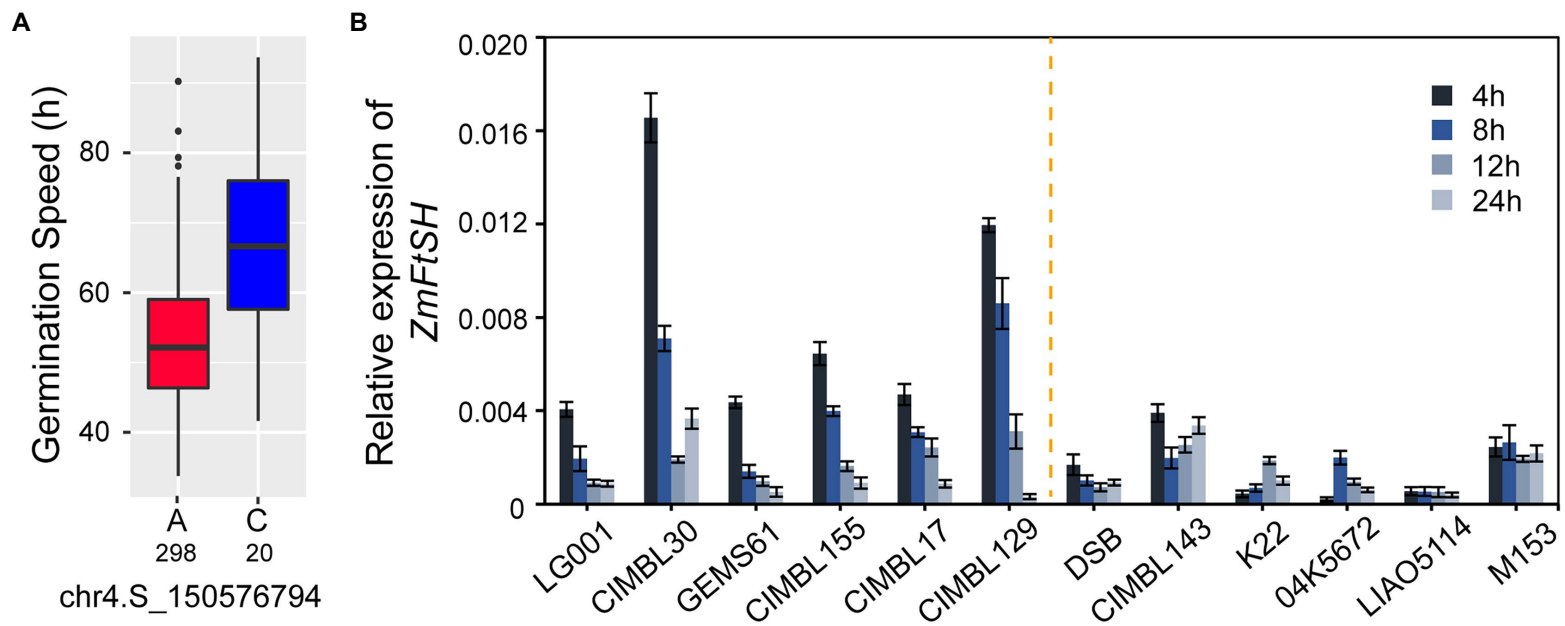
Allele effect of SNP and expression patterns of ZmFtsH during seed germination. **(A)** Allele effect of SNP chr4.S_150576794 for GS, 298 and 20 are represent the number of A and C genotype in population, respectively. **(B)** The relative expression level of ZmFtsH at seeds imbibition 4, 8, 12, 24 h by qRT-PCR in 12 lines, including LG001, CIMBL30, GEMS61, CIMBL155, CIMBL17, and CIMBL129, containing A allele; DSB, CIMBL143, K22, 04 K5672, LIA5114, and M153, containing C allele. Vertical bars indicate standard deviation.

GRMZM2G163193 contained the most significant SNPs of GC and GS (Figure 6A) and encoded the filamentation temperature sensitive H (FtsH) protease; accordingly, it was labeled as *ZmFtsH*. To further analyze the expression patterns of *ZmFtsH* between different genotypes, 12 inbreed lines were randomly selected from 321 lines based on the genotype of the gene’s most significant SNP. LG001, GEMS61, CIMBL30, CIMBL143, DSB, and K22 are all A allele genotypes for SNP chr4.S_150,576,794 that require less time for germination at the population level (SNP within *ZmFtsH*). In lines carrying the A allele genotype at SNP (chr4.S_150,576,794), the relative expression of *ZmFtsH* was higher during the initial stage of imbibition (4 h) but significantly decreased with a time extension. In contrast, the relative expression of *ZmFtsH* was lower in lines carrying the C genotype, and it remained relatively stable or increased gradually over time (Figure 6B).

### Alleles Pyramiding and Molecular Breeding Application

The allele effect of each independent significant SNP was investigated and shown in Supplementary Figure S1. GS values varied between alleles from 1.8 to 13.3 h. The SNP chr4.S_150,576,794 is the most significant locus (value of *p* = 2.04E-08, MLM) for GS, with the mean value of two alleles being 53.2 h (A allele) and 66.5 h (C allele; Figure 6A; Supplementary Figure S1). We also examined the allele effects of significant SNPs in GC and discovered that the effects between alleles of significant SNPs are significant (Supplementary Figure S2).

**Figure 7:**
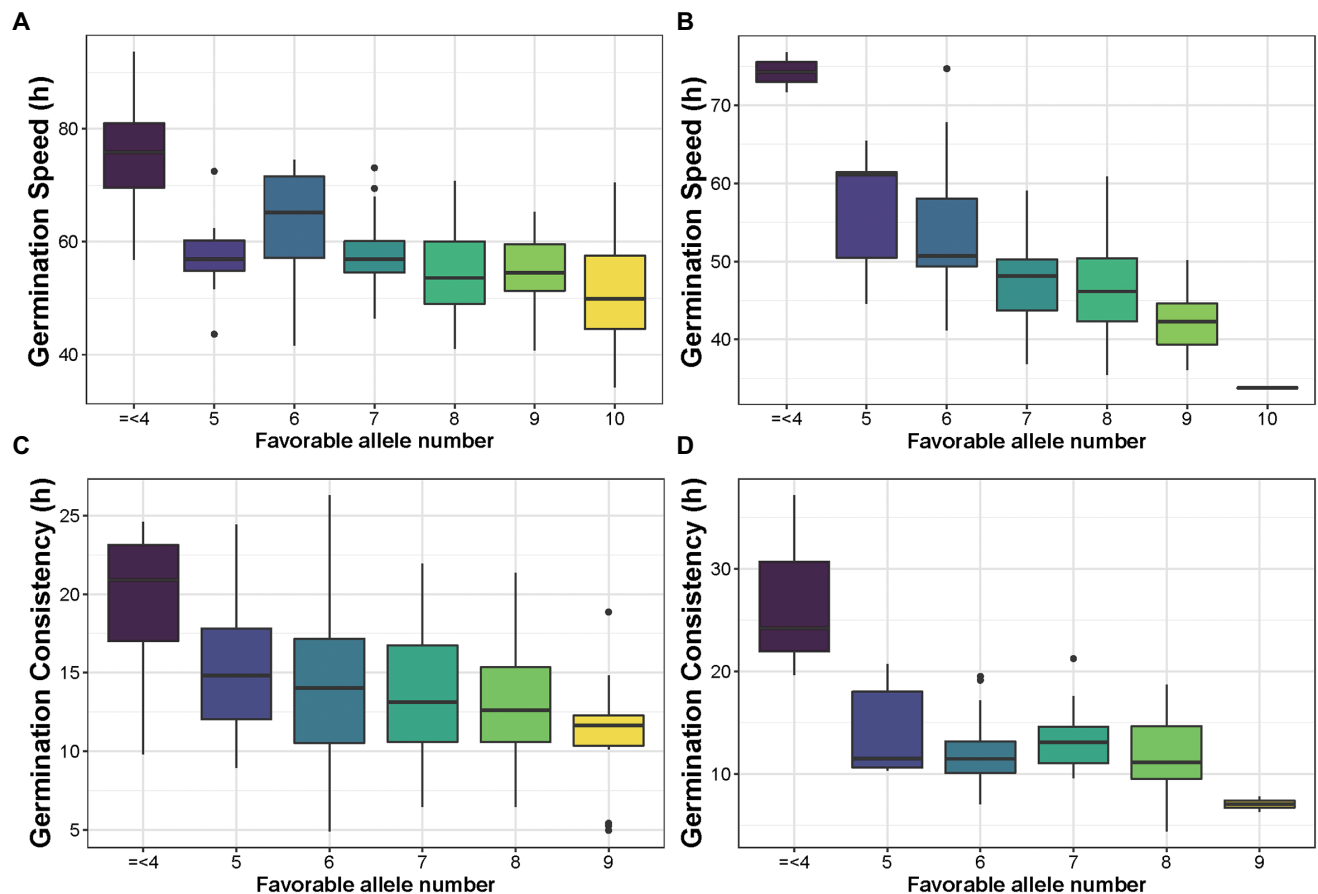
Boxplot of favorable alleles pyramiding for GS and GC in two germination traits. **(A)** Analysis of favorable alleles pyramiding for GS in the temperate subpopulation. **(B)** Analysis of favorable alleles pyramiding for GS in the tropical subpopulations. **(C)** Analysis of favorable alleles pyramiding for GC in the temperate subpopulation. **(D)** Analysis of favorable alleles pyramiding for GC in the tropical subpopulations.

**Figure 8:**
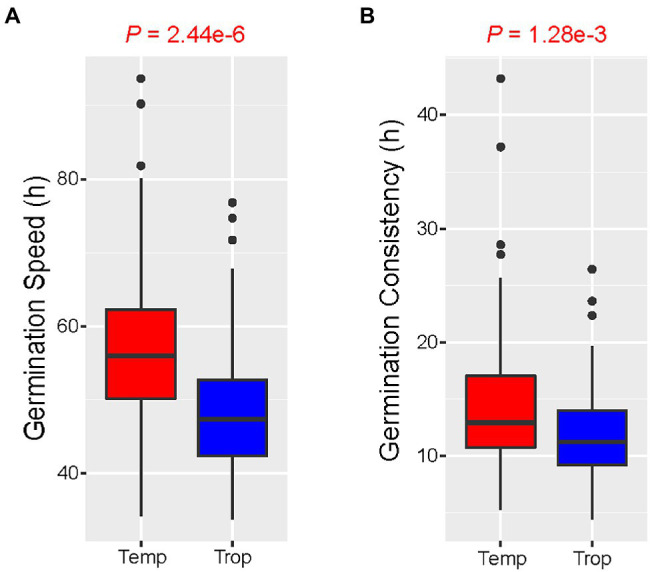
Boxplot of germination traits in a different subpopulation. **(A)** Analysis of variance to examine the difference in germination speeds between temperate and tropical subpopulations. **(B)** Analysis of variance to examine the differences in germination consistency between temperate and tropical subpopulations.

The 321 accessions used in this study were categorized into three subpopulations based on their population structure: temperate, tropical, and mixed. The favorable alleles are those that affect rapid or uniform germination. To demonstrate that favorable alleles can improve germination traits, allele pyramiding was used to determine the efficacy by increasing the number of favorable alleles. To avoid the effect of missing values, we used for further analysis only independent significant SNPs with a missing rate of less than 10% in each subgroup. As a result of the analysis, 10 SNPs were retained for GS and 9 for GC. With the accumulation of favorable alleles from GS, seed germination was observed to be faster, particularly in tropical populations. When the number of favorable alleles reached seven, GS gradually decreased in the temperate population (Figure 7A). In contrast, GS continued to decline as favorable alleles accumulated in the tropical populations (Figure 7B). Similar to the GS, seed germination became more uniform as the number of favorable alleles increased, particularly in tropical populations. When accessions carried more than five favorable alleles, germination uniformity was gradually increased in both temperate and tropical populations (Figures 7C,D). Furthermore, tropical lines germinate more rapidly and uniformly than temperate lines (Figures 8A,B). These results indicate that seed germination traits could be enhanced through molecular breeding by polymerizing these favorable alleles for cultivation requirements.

## Discussion

Seed germination is a very important trait in crop production. In previous studies, many indirect germination traits, such as germination percentage and seedling growth in germination Phase III, were used to evaluate the seed germination (Ji et al., 2009; Wang et al., 2010). However, seed germination Phases I and II are physiologically distinct from Phase III and are likely regulated by distinct regulatory pathways (Holdsworth et al., 2008). GS quantified the rate of germination and GC measured the uniformity of germination. Both traits provided an indirect insight into seed germination status during Phases I and II. Additionally, the rate and uniformity of germination were influenced by seed dormancy and environmental conditions during seed production. To reduce the noise in the germination tests, we used 321 maize accessions that had matured under similar environmental conditions to measure two seed germination-related traits (GS and GC) under -common germination conditions. Furthermore, all seeds used in the germination test had been dried and stored to break the dormancy. ermination phenotypes were counted every 4 h to increase accuracy.

Two germination traits exhibited a high degree of phenotypic variations, and the data showed normal distribution. Germination traits are complex and environment-dependent. The heritability (h2) was calculated to determine the number of genetic factors that contribute to phenotypic variation. The high h2 observed in GS and GC indicated that hereditary factors were the primary determinant of GS and consistency. A significant positive correlation was observed between GS and GC suggests that GS and uniformity were coordinated at population levels. On chromosomes 4, 5, 8, and 10, 12 common SNPs for GS and GC were identified, including the most significant chr4.S_150576794 for GS and the chr10.S_140984524 for GC (Figure 3). Two germination traits seem to be partially controlled by the same genetic factors.

Energy metabolism provides the energy required for seed germination. After several hours of imbibition, respiration is required to provide ATP *via* the tricarboxylic acid cycle and oxidative phosphorylation in the mitochondria (Hourmant and Pradet, 1981). Two FtsH proteins located in mitochondria have been shown to regulate the growth of Arabidopsis by influencing the activity of complexes involved in the oxidative phosphorylation pathway (Piechota et al., 2010). In this study, ZmFtsH (GRMZM2G163193) encoded an FtsH protein that was stored in dry seeds and expressed during seed germination. In the initial imbibition stage of 4 h, ZmFtsH expression was increased in SNP (chr4.S_150576794) A allele genotype lines (shorter germination time at population levels). The high expression of ZmFtsH contributes to energy mobilization and thus the seed germination in A allele genotype lines. The differential expression of ZmFtsH implies that the rate at which energy is mobilized may have important effects on germination traits in the natural population. PP2C played a role in and positively regulated seed germination (Brock et al., 2010). GRMZM2G141859 in SNP (chr8.S_133947794) encoded a PP2C-type gene that may be involved in the ABA signaling pathway that regulates seed germination. In a sense, seeds are stressed and cells are damaged during the maturation and drying processes. Certain signaling factors are activated, resulting in the production of protective proteins (Grelet et al., 2005; Tolleter et al., 2007). Calmodulin-like proteins (CMLs) function as the second massager in stress responses (Gao et al., 2004; Jiang et al., 2013). GRMZM2G129133 encoded a CML protein, which is expressed during seed maturation and germination.

PTMs of proteins affect their stability, activity, and function. Proteins that were damaged and lost function during maturation, death, and imbibition were repaired by PTMs. A series of protein kinases and phosphatases involved in the regulation of proteins phosphorylation and dephosphorylation have been implicated in seed germination regulation (Brock et al., 2010; Hubbard et al., 2010). It has been proposed that phosphorylation is involved in DNA repair during seed germination (Lu et al., 2008). In this study, four genes related to phosphorylation and dephosphorylation were identified, including three protein kinase genes (GRMZM2G137788, GRMZM2G059893, and GRMZM5G882078) and one phosphatase gene (GRMZM2G141859). Additionally, an acyltransferase gene (GRMZM2G098079) was identified as a candidate gene for seed germination. Gene expression is regulated on multiple levels by regulatory factors during germination, for example, RNA transcription initiation and RNA processing (Lee et al., 2015; Guo et al., 2018b). We identified a transcription factor (GRMZM2G063387) and an RNA-binding protein (GRMZM5G841101) as potential regulators for germination.

Germination traits vary among maize subpopulations at the population level, and seed germination takes less time in tropical accessions than in temperate accessions. We analyzed alleles from different subpopulations and found that favorable alleles accumulated in GS and GC had a greater effect on the tropical subpopulation than on the temperate subpopulation. Seed germination is different from seed dormancy. When seed dormancy is broken, tropical accessions germinate more rapidly than temperate accessions. Seed must likely germinate immediately under suitable conditions to avoid the invasion of bacteria and viruses in tropical regions with high temperatures and humidity. Therefore, more attention should be paid to the tropical germplasm resources for seed germination. GC of tropical subpopulations is also better than temperate subpopulations but not as significantly as GS, which may be due to shorter germination times in tropical subpopulations demonstrating more uniform germination than temperate subpopulations. This finding implies that the differences in GC between two subpopulations may be due to factors other than genetics.

In conclusion, the germination traits of maize seeds were critical for production. During the breeding process, traits such as rapid and uniform germination that are suitable for human needs are selected. Almost all significant SNPs in our study showed distinct phenotypes between alleles. With an increase in the number of favorable alleles (except for very much or very little), germination became more rapid and uniform (Figure 7). GWAS was used to dissect the seed germination traits in the 321 maize inbred lines. Furthermore, 58 significant SNPs were identified, reflecting the complexity of maize seed germination. Several candidate genes that were associated with significant SNPs were identified, and one of them, the energy metabolism-related gene *ZmFtsH* (GRMZM2G163193), may be more important for seed germination in maize. These results suggested that using SNPs derived from natural variation as markers will be beneficial for future molecular breeding of maize seed germination.

## Data Availability Statement

The original contributions presented in the study are included in the article/Supplementary Material; further inquiries can be directed to the corresponding author.

## Author Contributions

CL and YaL: conceptualization and methodology. YuL, QZ, JY, ML, and ZW: data collection. YaL and ML: formal analysis. CL: funding acquisition. JF and YR: investigation. CL and AZ: supervision. YaL and XD: visualization. YuL, CL, and YaL: writing—original draft. All authors contributed to the article and approved the submitted version.

## Funding

This work was supported by National Nature Science Foundation of China (no. 31601233) and National Foreign Experts Program of China (no. DL2021006002L).

## Conflict of Interest

The authors declare that the research was conducted in the absence of any commercial or financial relationships that could be construed as a potential conflict of interest.

## Publisher’s Note

All claims expressed in this article are solely those of the authors and do not necessarily represent those of their affiliated organizations, or those of the publisher, the editors and the reviewers. Any product that may be evaluated in this article, or claim that may be made by its manufacturer, is not guaranteed or endorsed by the publisher.
