# Supplementary material for: Genome-Wide Association Studies Provide Insights Into the Genetic Architecture of Seed Germination Traits in Maize
Source: Front Plant Sci. 2022 Jun 10;13:930438. doi: 10.3389/fpls.2022.930438 (PMC9226777; doi:10.3389/fpls.2022.930438)
Supplement: Supplementary file 1 [file Data_Sheet_1.docx]

Genome-wide association studies provide insights into the genetic architecture of seed germination traits in maize

Yuntong Li^1†^, Yameng Liang^2†^, Meiling Liu^1^, Qiyuan Zhang^1^, Ziwei Wang^1^, Jinjuan Fan^1^, Yanye Ruan^1^, Ao Zhang^1^, Xiaomei Dong^1^, Jing Yue^3^ and Cong Li^1^*

^1^ College of Bioscience and Biotechnology, Shenyang Agricultural University, Shenyang 110866, China.

^2^ State Key Laboratory of Plant Physiology and Biochemistry, National Maize Improvement Center, Key Laboratory of Biology and Genetic Improvement of Maize (MOA), Beijing Key Laboratory of Crop Genetic Improvement, China Agricultural University, Beijing 100193, China.

^3^ College of Pharmaceutical and Biological Engineering, Shenyang University of Chemical Technology, 110866, China.

* Correspondence: lnlicong_123@syau.edu.cn

† These authors have contributed equally to this work and share first author ship.


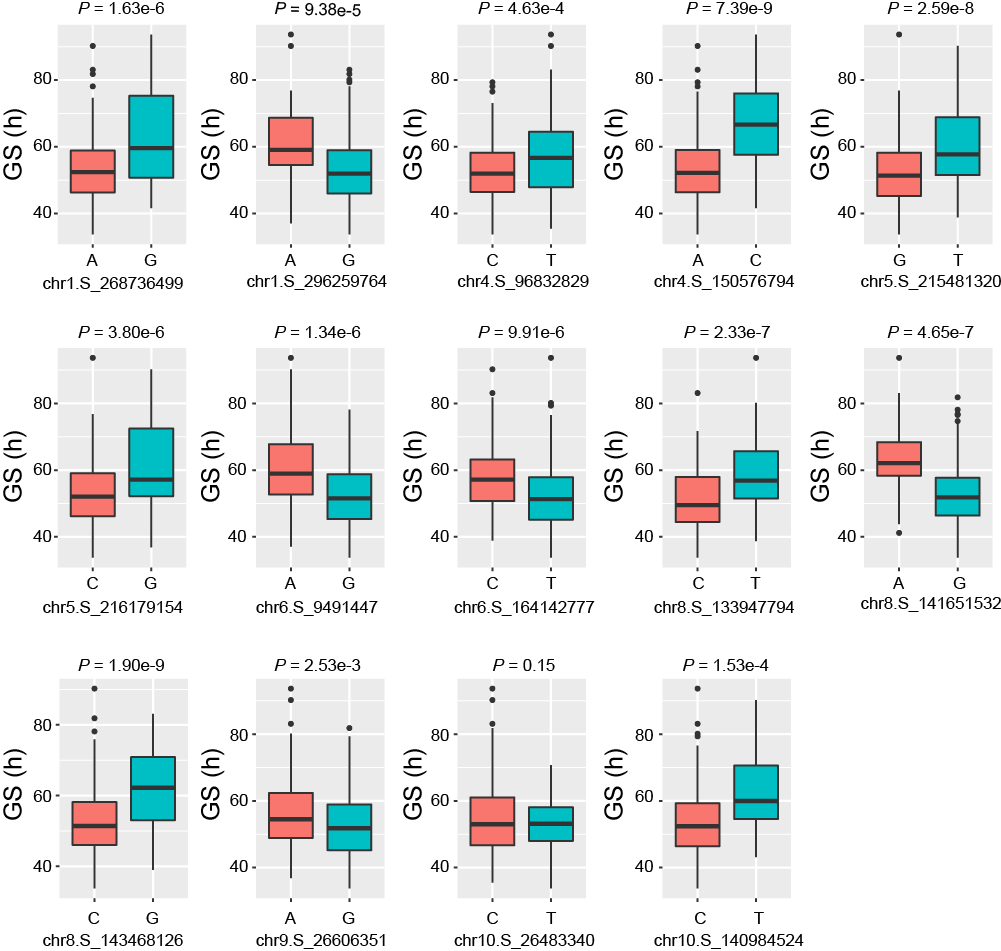


Figure S1 Allele effects of the significant SNPs for germination speed.


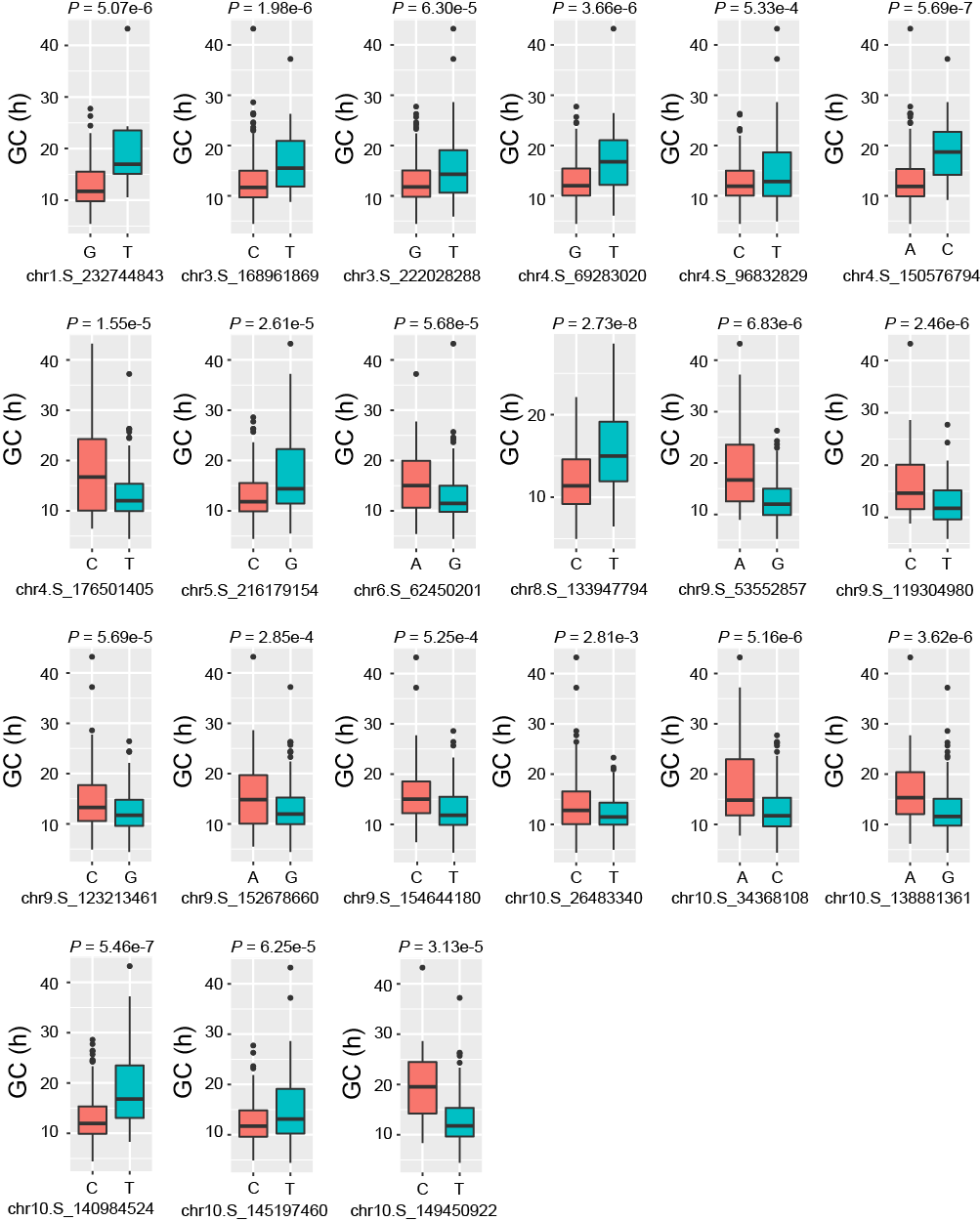


Figure S2 Allele effects of the significant SNPs for germination consistency.

Table S1 Sequence of the primers used in this study

| **Gene name** | **Primer mane** | **Primer sequence (5'-3')** |
| --- | --- | --- |
| *ZmTubulin1* (*Zm00001d013367*) | ZmTubulin1-F | GTGTCCTGTCCACCCACTCTCT |
|  | ZmTubulin1-R | GGAACTCGTTCACATCAACGTTC |
| *GRMZM2G163193* | FTSH-F | TGGCGAGCATAGACAAGGACC |
|  | FTSH-R | CCAGCATTATCGACGACAGCA |
| *GRMZM2G059893* | 059893-F | CGCAACCACAACCATCACTA |
|  | 059893-R | GACAGCCTTCCAGAACCATT |
| *GRMZM2G129133* | 129133-F | GTCTTCTCCGTCGGGCTCAT |
|  | 129133-R | CGAGGCAGTGAAAGCGAAAC |
| *GRMZM2G063387* | 063387-F | AAGGAAATGCGGTTCTGTGG |
|  | 063387-R | CCCTGGAATAGGCGGTGAGC |
| *GRMZM2G098079* | 098079-F | TGGGTTGGGTGAACGGTGAA |
|  | 098079-R | AGAGGCGCAGGAATGGAAGC |
| *GRMZM2G109987* | 109987-F | CCCCAACAACTTTAGTCCCT |
|  | 109987-R | AAGCATCTCAGCCCTAACAA |
| *GRMZM5G841101* | 841101-F | CTACGGCTTCGTCTGCTACTCC |
|  | 841101-R | TCGCATAGGCATCCATCACAT |
| *GRMZM5G869572* | 869572-F | ATCAGGATGGTTGGTTGGGTC |
|  | 869572-R | CACCTGCTAAGAGGAAGAGGAAGA |
